# Promyelocytic Sarcoma of the Spine: A Case Report and Review of the Literature

**DOI:** 10.1155/2010/137608

**Published:** 2010-03-18

**Authors:** Leonardo Pacilli, Francesco Lo Coco, Safaa Mahmoud Ramadan, Laura Giannì, Alberto Pingi, Daniele Remotti, Ignazio Majolino

**Affiliations:** ^1^Hematology and BMT Unit, Institute of Hematotherapy, Ospedale S. Camillo, 00153 Rome, Italy; ^2^Department of Biopathology, Tor Vergata University, 00133 Rome, Italy

## Abstract

Myeloid sarcoma (MS, previously named granulocytic sarcoma or chloroma) is a rare extramedullary tumour of immature myeloid cells. It can be present before, concurrently with, or after the diagnosis of acute myeloid leukemia. MS is extremely uncommon in acute promyelocytic leukemia (APL). In the case described here, MS was the sole site of APL relapse and the cause of spinal cord compression. The patient presented with neurologic symptoms due to a paravertebral mass of MS after 7 years of complete remission. He was treated with excision of the mass followed by local radiotherapy. Systemic treatment was also given with combined arsenic trioxide and all-trans retinoic acid and the patient was able to achieve a second prolonged clinical and molecular remission.

## 1. Introduction

Myeloid sarcoma (MS, previously named granulocytic sarcoma or chloroma) is a rare extramedullary tumour of immature myeloid cells [1]. It can occur in association with myelogenous leukemia, myeloproliferative disorders, and myelodysplasia. MS has different modalities of presentation and can affect any organ. It may occur otherwise in healthy individuals who subsequently develop an overt typical myelogenous leukemia [1, 2]. MS can also develop in leukemia patients concurrently with or after diagnosis, or as a manifestation of disease relapse [3, 4]. The WHO recognizes three major variants of MS based on the predominant cell type and the degree of maturation, namely respectly, a myeloblast variant, with a mix of myeloblasts and promyelocytes variant, and more differentiated variant with promyelocytes and more mature granulocytes [5]. Acute promyelocytic leukemia (APL) accounts for approximately 10% of acute myeloid leukemia (AML) and cases of MS due to APL have been only occasionally reported [1–4]. We report the case of a patient with APL who presented right paraparesis after 7 years of complete remission. The patient had a MS with paravertebral localization as the only site of APL relapse.

## 2. Case Report

**Figure 1:**
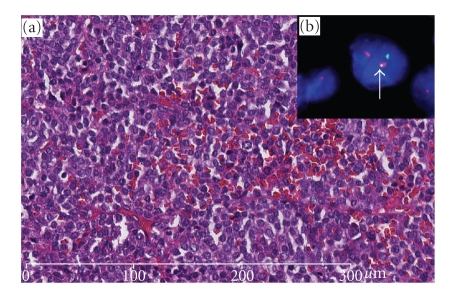
(a) The pictures show adipose and fibrous tissue with dense and diffuse infiltrate of monomorphic PML cells (H\E, 20×). (b) FISH revealed the presence of the reciprocal and balanced t(15;17) on PML cells (arrow).

In November 1999, a 38-year-old male presented with a history of recurrent episodes of gum bleeding. Physical examination was unremarkable, but a complete blood count showed Hb 6.1 g/dL, WBC 1 × 10^9^/L, and platelets 2 × 10^9^/L. Microscopic examination of a blood smear showed 8% hypergranular promyelocytes, with Auer rods. At examination of bone marrow aspirate a diffuse infiltration by atypical promyelocytes was apparent. The immunophenotype profile of leukemic cells was consistent with APL (CD33+ve, CD45+ve, HLA-DR-ve, CD34-ve, CD16-ve, and CD56-ve). Cytogenetics revealed the characteristic t(15;17) (q22;q21-q22) and the RT-PCR assay showed the typical PML/RAR*α* fusion gene thereby confirming the diagnosis of APL [6]. The patient was treated with all-trans retinoic acid (ATRA) 45 mg /m²/day ber 30 days and idarubicin (IDA) 12 mg/m²/day on days 2, 4, 6, and 8. On day 20, the patient had severe pulmonary distress that was consistent with an ATRA syndrome [7]. This was successfully managed with high-dose dexamethasone and ATRA discontinuation. Despite that, following induction, bone marrow evaluation revealed a morphologic and molecular picture of complete remission. The patient received 3 additional courses of chemotherapy as consolidation and completed his treatment in July 2002. Five years later in July 2007, the patient presented with right chest pain and progressive right paraparesis in a few-week duration. An MRI of the spine revealed a solid extramedullary intraspinal mass extending between T6 and T8. Diagnostic workup including coagulation profile and complete blood counts was normal, and neither atypical promyelocytes nor blast cells were detected in peripheral blood and bone marrow. In addition, PCR analysis for PML/RAR transcripted on bone marrow cells confirmed a status of molecular remission. Cord decompression was performed with total laminectomy and excision of an extradural soft tissue mass of 4 cm × 5 cm × 5 cm. Histopathology examination showed diffuse infiltration by monomorphic, medium-sized, neoplastic cells with round shaped deeply indented or bilobated nuclei with dispersed chromatin, inconspicuous nucleoli, and basophilic cytoplasm. The immunophenotype characteristics of neoplastic cells were as follows: myeloperoxidase and CD117 (c-KIT) were strongly positive; CD45 was weakly and focally expressed while CD34, CD31, CD20, CD79a, CD3, CD56, and CD15 were negative. Cytogenetic studies showed (Figure 1) t(15;17) (q22;q21-q22) with PML/RAR*α* fusion gene as demonstrated by FISH LSI with PML/RAR*α* probe dual color (Dual fusion translocation probe, Vysis/Abbot). Since the disease was localized to the spine, the patient underwent a local radiotherapy with 36 Gy.

Systemic therapy was also started on an outpatient basis with combined ATRA and arsenic trioxide (ATO) for a total of 5-month cycles according to the scheme reported by Estey et al. [8]. The patient were able to achieve and maintain a second complete hematologic remission. Currently, he is still in remission and well 20 months since the end of treatment.

## 3. Discussion

Extramedullary disease (EMD) at diagnosis or at relapse develops in 3%–8% of patients with acute myelogenous leukemia, more frequently in those with myelomonocytic and monocytic morphology [9–11] (M4 and M5 French- American- British subtypes). Its occurrence in APL is relatively rare but after the advent of ATRA it is increasingly reported at time of relapse [11–29]. The case reported here, is a rare case of CNS isolated relapse [13]. The patient presented with neurologic symptoms due to a paravertebral mass after 7 years of complete remission. He entered a second prolonged clinical and molecular remission after local radiotherapy followed by a combination of ATRA and ATO. This case represents the second report of MS resulting in spinal cord compression due to APL extramedullary relapse. The other case, reported by Tsimberidou et al. [30], was a man who remained in complete remission for 3 years after treatment of APL. This patient later developed paraplegia due to a paravertebral mass at the level of T7-T8. Following decompression laminectomy, an isolated extramedullary promyelocytic relapse was diagnosed. The bone marrow was not involved. The patient was treated with local radiotherapy followed by ATRA and idarubicin, achieving a short second remission. He subsequently developed a second relapse with marrow and skin involvement. 

 The occurrence of EMD has long been considered a rare event in APL patients treated with chemotherapy alone, whereas this phenomenon has increasingly been reported in the ATRA era [5, 9, 38]. The question arises as to whether treatment of APL with ATRA predisposes patients to the development of EMD [38, 51]. This question is still open, as various trials have shown contrasting results. Wiernik et al. [3] suggested that extramedullary APL occurs more frequently after ATRA than other therapies. Ko et al. [23] demonstrated that patients receiving ATRA induction had a 2.1 increased relative risk of EMD compared with those with chemotherapy alone. In a literature review by Bae et al. [51], only three of 21 cases with CNS relapse received systemic chemotherapy without ATRA. Among 172 patients with APL treated at MDACC between 1980 and 2003, a total of three patients relapsed with isolated EMD and it occurred exclusively in patients who had received ATRA-containing induction regimens [51]. Ohno et al. [52] noted that EM relapse was absent from all 37 patients with relapsing APL in the Japanese chemotherapy only studies, while it was seen in 8% of 121 patients in the chemotherapy plus ATRA protocols and this raised the possibility that the doses of chemotherapy given with ATRA-based regimens are less intensive than those previously used and thus may not reach therapeutic levels in the EM tissues. This could be particularly relevant to protocols without cytarabine. A relationship between EMD and ATRA treatment has been suggested. Two possible explanations are considered. The first suggests a direct effect of ATRA on adhesion molecules resulting in increased infiltration capability of APL leukemia blasts [23]. The second postulates the occurrence of MS in relapsed patients as a consequence of the prolonged survival [38]. In a study of the GIMEMA [38], the authors found no statistical difference in the frequency of extramedullary involvement between patients treated with or without ATRA. EMD was documented in 5% and 12% of patients included in the LAP0389 and AIDA protocol, respectively, with no statistical difference. In particular, the five EMD localizations in the LAP0389 study were in the CNS (1 patient), the skin (3 patients), and the middle ear (1 patient). Of the 16 EMD relapses in the AIDA study, 10 involved the CNS and six involved other extramedullary sites (the skin in 3, the middle ear in 2, and the lung in 1). Thus CNS disease at relapse seems frequent among the AIDA treated patients (8% versus 1%) [39]. Breccia et al. [50] described three adult patients with middle ear localizations at relapse. They had been previously treated with ATRA and CT. Their characteristics are shown in Table 1.

Similarly, C. Samanez et al. [53] reported 7 cases of EMD relapse in APL patients receiving chemotherapy (CT) alone (102 patients) or combined with ATRA (155 patients). EMD relapse rate was 4.4% in the CT group and 8.7% in the group CT plus ATRA, with no statistical differences. EMD localizations were in soft tissues (3 patients), the CNS (2 patients), the tonsils (1 patient), liver (1 patient), and the testis (1 patient). De Botton et al. [54] analyzed EMD relapse occurring in patients with APL treated with ATRA and CT. Of 740 patients included in three multicenter trials (APL91, APL93 trials, and PETHEMA 96) 10 patients developed EMD relapse. Of these, 9 were in the CNS and one in the skin. Only two patients had isolated EMD relapses. A significant correlation could be found between high WBC count and the risk of CNS relapse. More recently, Casanova et al. [55] reported a similar pattern of increased CNS EMD relapse in APL patients treated with ATRA and CT. EMD relapse was found in 8 of 74 (15%) APL patients (CNS: 5, external ear: 3). Together, these studies confirm that the CNS is the preferential site of extramedullary involvement in APL, raising an issue of whether or not to consider CNS prophylaxis in APL treatment protocols especially in patients presenting with hyperleukocytosis, as suggested by the expert panel of the European Leukemia Net. However, the benefit of this policy has not been established in prospective studies [56].The occurrence of the ATRA syndrome was recognized to be a significant risk factor for EMD involvement at relapse [57]. Since APL cells, in patients affected by ATRA syndrome, infiltrate multiple tissues and organs, including the lung and the skin, it was hypothesized that ATRA could promote the migration of differentiating blasts into the skin, the CNS, and other tissues which represented a reservoir of viable blasts. These cells could later proliferate and result in an EMD recurrence [9, 11, 23, 27, 57]. Others have found that elevated WBC count (>10 × 10^9^ L) on presentation [55], predominance of the PML/RAR*α* bcr3 type, and mirogranular morphology [50] are considered as risk factors. 

 Management of relapse in the CNS and other extramedullary sites in patients with APL is a challenging issue for which there is a notable lack of information. EMD relapse, including in the CNS, can occur either in isolation or associated with BM involvement as a first relapse, and also after one or more hematologic relapses. Optimal management and outcome of APL patients in these different situations have not been critically assessed. In case of granulocytic sarcoma, wherever it is localized, radiation and intensive systemic therapy might be considered. 

 Before the demonstration of the striking activity of ATO in APL, salvage therapy usually consisted of the readministration of ATRA and chemotherapy for induction, generally containing high-dose cytarabine and an anthracycline, followed by further chemotherapy and/or hematopoietic stem cell transplantation (HSCT) [58–60]. Confirmation of the high and sustained efficacy of ATO in patients with relapsed/refractory APL has been provided by more recent studies [61, 62]. CR rates in these trials were 80%–90% and, in those studies that evaluated survival, 50%–70% of patients were alive at 1 for 3 years. Current evidence suggests that the use of at least 2 cycles of ATO results in the achievement of second molecular CR in nearly 80% of cases [63]. The best consolidation strategy after ATO induced second remission is unknown; options include continued treatment with repeated cycles of ATO, the use of standard chemotherapy in combination with ATRA and/or ATO, and HSCT. For patients unfit to proceed to HSCT, the available options include repeated cycles of ATO with or without ATRA/standard chemotherapy. 

 The central nervous system (CNS) is the commonest site of extramedullary disease in APL and at least 10% of hematologic relapses are accompanied by CNS involvement [11]. Because the majority of CNS relapses occur in patients presenting with hyperleukocytosis some strategies include CNS prophylaxis for patients in this particular high-risk setting. For such patients, it is advisable to postpone CNS prophylaxis until after the achievement of CR because lumbar puncture at presentation and during induction is extremely hazardous. However, the benefit of this policy has not been established. For patients without hyperleukocytosis, in whom the risk of CNS relapse is extremely low, there is a general consensus to avoid CNS prophylaxis. 

 CNS prophylaxis for patients in this particular high-risk setting. For such patients, it is advisable to postpone CNS prophylaxis until after the achievement of CR because lumbar puncture at presentation and during induction is extremely hazardous. However, the benefit of this policy has not been established. For patients without hyperleukocytosis, in whom the risk of CNS relapse is extremely low, there is a general consensus to avoid CNS prophylaxis. 

 In conclusion, the increasing number and special pattern of EMD involvement in relapsing APL patients emphasize its importance in the differential diagnosis of EMD localization in patients with history of APL. they also highlight the need for understanding the underlying pathogenesis and predisposing factors, as well as for selecting the optimal treatment approach.

## Figures and Tables

**Table 1:** Clinical features of patients with MS in APL.

References	No. cases	disease status	Time since	Site of involvement
			diagnosis/EMD relapse (months)	
Fukushima et al. [31]	1	onset		cerebellum + hematological
Worch et al. [32]	1	onset		lytic lesions of humerus, tibia, femur + molecular hematological
Ajarim et al. [33]	1	onset		thymus + hematological
Savranlar et al. [34]	1	onset		thoracic-epidural
Brown et al. [35]	1	onset		optic nerve + hematological
Agarwal et al. [36]	1	relapse	31	hip + hematological
Disel et al. [37]	1	relapse	9	pleura
Tsimberodou et al. [30]	1	relapse	36	thoracic spine
Specchia et al. [38]	1	relapse	NA	lung + hematological
	3	relapse	155	mastoid + hematological
Latagliata et al. [39]			71	mastoid + mol hematological
			61	mastoid
	3	relapse	7	subcutaneous nodules at sternal manubrium + wrist at level of the radial artery pulse + site of intravenous catheter scar+ mol hematological
Sanz et al. [40]			6	subcutaneous nodules at jugular catheter scar + wrists at level of the radial artery pulse + molecular hematological
			32	sternal manubrium + subcutaneous nodule + antecubital fossa
Magliulo et al. [41]	1	relapse	24	external auditory canal+ mol hematological
Nasilowska-Adamska et al. [42]	1	relapse	21	pleura, heart and pericardium
Slavecheva et al. [43]	1	relapse	120	lymph node
Kai et al. [44]	1	relapses	NA	4 time at different sites
Tobita et al. [45]	1	relapses	NA	1st and 2nd external auditory canal
Forrest et al. [46]	1	relapse	1st 48-2nd 24	1st testicular -2nd retroperitoneal nodes, psoas muscle and skin
Skarin et al. [47]	1	relapse	8	CNS + hematologic
Leoni et al. [48]	2	relapse	NA	(1) central nervous system + middle ear + lymph nodes.
				(2) skin + lymph nodes
Ammatuna et al. [49]	1	relapse	NA	1st and 2nd -scalp + mol hematological 3rd mol hematological + breast
Breccia et al. [50]	7	relapse	5 from CR	(1) left auricular canal + mastoid + mol hematological
			69	(2) left mastoid + mol hematological
			58	(3) left mastoid
			9	(4) left mastoid + hematologic
			12	(5) left mastoid + hematologic
			64	(6) right auricular canal
			6 from 2nd CR	(7) right mastoid + CNS + mol hematological

NA: not available; mol: molecular; CR: complete remission;
